# Development of Biodegradable Foam Trays from Brewer’s Malt Bagasse and Potato Residues from Agricultural Crops

**DOI:** 10.3390/polym17152146

**Published:** 2025-08-06

**Authors:** Evelyn F. Vásquez-Bacilio, Cesar I. Mejia-Llontop, Carlos E. Tirado-Rodríguez, María de Fátima Arévalo-Oliva, Beetthssy Z. Hurtado-Soria, Eudes Villanueva, Gilbert Rodriguez, Delia Rita Tapia-Blácido, Elza Aguirre

**Affiliations:** 1 Escuela de Posgrado, Universidad Nacional del Santa, Av. Universitaria s/n, Nuevo Chimbote 02712, Ancash, Peru; 2022740001@uns.edu.pe; 2 Laboratorio de Microbiología y Toxicología de Productos Agroindustriales, Universidad Nacional del Santa (UNS), Urb. Av. Universitaria s/n, Nuevo Chimbote 02712, Ancash, Peru; 201023040@uns.edu.pe (C.I.M.-L.); 2024818024@uns.edu.pe (M.d.F.A.-O.); 3 Departamento Académico de Ingeniería en Industrias Alimentarias, Universidad Nacional Autónoma de Tayacaja Daniel Hernández Morillo (UNAT), Jr. Bolognesi Nro. 418, Pampas 09156, Tayacaja, Peru; beetthssy.hurtado@unat.edu.pe (B.Z.H.-S.); eudesvillanueva@unat.edu.pe (E.V.); 4 Facultad de Ingeniería, Universidad Nacional del Santa (UNS), Urb. Av. Universitaria s/n, Nuevo Chimbote 02712, Ancash, Peru; grodriguez@uns.edu.pe; 5 Department of Chemistry, Faculty of Philosophy, Sciences and Letters of Ribeirão Preto, University of São Paulo, Av. Bandeirantes, 3900, Ribeirão Preto 14040-901, Brazil; delia@ffclrp.usp.br

**Keywords:** *Solanum tuberosum*, amylopectin, fiber, spectroscopy, bioeconomy

## Abstract

In light of the environmental impact of disposable products made from petroleum-based plastics, this study focused on developing biodegradable foam trays made from a starch (PS) derived from potato waste and beer malt flour (BMBF). The objective of this study was to evaluate the effect of the concentration of BMBF on the physical and mechanical properties of potato starch-based foam trays prepared by the thermoforming process at temperatures of 150 °C (upper plate) and 145 °C (lower plate) for 5 min and 40 s. The results showed that increasing the BMBF concentration from 0 to 40% reduced the moisture content from 4.68% to 3.42%, increased the thickness from 2.63 cm to 4.77 cm, and decreased the density from 0.28 g.cm^−3^ to 0.15 g.cm^−3^. Meanwhile, the water absorption capacity increased from 38.7% to 69.7%. In terms of mechanical properties, increasing the BMBF concentration in the PS foam tray resulted in a decrease in hardness from 5.61 N to 2.87 N, a decrease in tensile strength from 2.92 MPa to 0.85 MPa, and a decrease in elongation from 1.42% to 0.59%. Meanwhile, fracturability increased from 2.04 mm to 3.68 mm. FTIR analysis revealed interactions between BMBF and PS in the composite foam tray. Thermogravimetric analysis (TGA) showed two thermal events: one between 20.96 °C and 172.89 °C, and another between 189.14 °C and 517.69 °C, with weight losses of 5.53% and 74.23%, leaving an ash residue of 20.24%. Differential calorimetry analysis (DSC) showed a glass transition at 152.88 °C and a melting at 185.94 °C, with an enthalpy of fusion of 74.11 J.g^−1^. Higher concentrations of BMBF (>10%) decreased the water resistance, mechanical strength, and flexibility of the PS foam trays. Therefore, a formulation of 90% PS and 10% BMBF was better for producing a foam tray with improved mechanical properties and water resistance, which could be used as a sustainable alternative to conventional single-use plastic.

## 1. Introduction

The massive use of plastics has grown exponentially in recent decades, consolidating as an essential material in various sectors due to its low cost, flexibility, and ease of use [1]. However, this popularity has brought with it serious environmental and public health consequences. Plastics, mostly derived from petroleum, are resistant to water, sun, and other environmental factors, making them difficult to degrade rapidly. This makes them one of the main sources of persistent pollution in the environment [2]. Microplastics, fragments smaller than five millimeters that originate from the erosion of larger plastics, negatively affect ecosystems and human health, as they are ingested by marine organisms and accumulate in the food chain. In addition, they serve as vehicles for invasive species and adsorb pollutants such as PCBs (Polychlorinated Biphenyls), PAHs (Polycyclic Aromatic Hydrocarbons), and DDT (Dichlorodiphenyltrichloroethane), increasing their toxicity due to the plasticizers and heavy metals they contain [3].

In 2010, 275 million metric tons (MT) of plastic waste were generated in 192 coastal countries, of which between 4.8 and 12.7 million MT entered the ocean [1]. In 2021, global plastics production exceeded 400 million t, and it is estimated that, without effective interventions, this figure could increase to 590 million by 2050 or even triple to more than 1.2 billion t by 2060, according to projections in the OECD’s Global Plastics Outlook report [4]. Microplastics in the oceans have increased, reaching up to 30 kg km^−2^ in some areas [5]. In Peru, Supreme Decree N°006-2019-MINAM and Law N°30884 regulate single-use plastics to mitigate their environmental and health impacts (MINAM, 2019) [6]. It has been shown that synthetic plastics can take 100 to 500 years or more to fully degrade [7]. In contrast, biodegradable materials, such as those developed from organic waste, have much shorter degradation times, ranging from six months to one year [8]. However, some commercial bioplastics such as polylactic acid (PLA) face economic and technical limitations, as they require specific industrial composting conditions such as high temperatures and controlled humidity to degrade properly, which reduces their effectiveness as a truly sustainable alternative [9].

This makes them a viable and environmentally responsible alternative to replace conventional plastics. The agro-industrial and agricultural sectors in Peru offer an opportunity to address the organic waste issue. The brewing industry generates 15 million kg of malt waste (bagasse) annually, most of which is disposed of on wasteland, decomposing inappropriately and releasing methane, a greenhouse gas responsible for 30% of global warming [10]. Potato cultivation, which is important for the Peruvian economy, generates 50 million t of waste per year, mainly from unharvested potatoes that are burned along with the stubble. This process not only pollutes the air but also wastes resources that could be reused for productive purposes. In addition, potato residues in the food industry, such as those from compotes and snacks, remain underutilized [11]. However, these residues contain starch that could be recovered for use in producing bio-based materials. Although biodegradable alternatives to petroleum-derived plastics have been studied, the combination of brewer’s spent grain and potato starch remains largely unexplored, particularly in thermoformed applications [12]. Brewer’s spent grain is rich in lignocellulosic fiber and proteins [13], while potato starch, known for its film-forming and biodegradable properties [14], offers complementary characteristics that could enhance the strength and degradability of bioplastics. This study proposes a synergistic formulation of these two agro-industrial residues, evaluating their effects on the physical, mechanical, and thermal properties of thermoformed foam trays. Unlike previous studies, this research incorporates advanced characterization techniques such as Fourier Transform Infrared Spectroscopy (FTIR), thermogravimetric analysis (TGA), and differential scanning calorimetry (DSC) to investigate molecular interactions and thermal transitions. This type of research aligns with the Sustainable Development Goals (SDGs), in particular SDG 12 (responsible production and consumption), SDG 13 (climate action) and SDG 14 (underwater life), promoting a circular economy that reduces pressure on natural ecosystems and encourages the responsible use of resources [15,16].

The study gains significant environmental relevance by valorizing millions of tons of agro-industrial waste generated both in Peru and globally [6,17], fostering a circular bioeconomy through low-cost solutions with potential for sustainable packaging across various sectors. This is in line with recent studies that emphasize the urgent need to develop bioplastics from agricultural residues and evaluate their environmental impact as alternatives to conventional plastics [18]. This approach not only aims to reduce reliance on single-use plastics but also adds value to organic waste by promoting its reuse in the production of new materials.

## 2. Materials and Methods

### 2.1. Raw Materials and Reagents

Potato residues of the Ismena variety (yellow pulp) were obtained from 69 organized farmers in the district of Carabamba, in the province of Julcan, La Libertad, Peru. The farmer reported an average yield of cultivated potatoes of 13.3 t per hectare. Of this amount, approximately 3% was left unharvested, equivalent to 0.4 t of stubbled potatoes per hectare. For the project, a sample of 30 kg of potato stubble, representing 7.5% of the total generated, was selected for starch extraction. The brewing malt bagasse was provided by the Microbiology and Toxicology Laboratory of the Faculty of Agroindustrial Engineering of the Universidad Nacional del Santa (UNS), Nuevo Chimbote, Peru, as a by-product of the craft beer brewing process and consisted of Pale Ale malt of the commercial brand Bestmalz (Heidelberg, Germany), whose characteristics were: Plant impurities/foreign grain: max. 1%; Foreign matter (metal/glass): absent; No plant particles: max. 0.1%; Mycotoxins (Aflatoxin B1: max. 2 μg.kg^−1^, Aflatoxin B1 + B2 + G1 + G2: max. 4 μg.kg^−1^, Ochratoxin: max. 3 μg.kg^−1^, Zearalenone: max. 100 μg.kg^−1^, Deoxynivalenol (DON): max. 750 μg.kg^−1^); Heavy metals (Lead: max. 0.2 mg.kg^−1^ and Cadmium: max. 0.05 mg.kg^−1^); The malt was free of ionization and radiation. The barley malt was immersed in 40 L of water at 75 °C for 1 h. The water was collected, and then 30 L of additional water at 75 °C was added. After 10 min, this water was also collected. The objective of this process was to remove as many carbohydrates as possible from the malt for the brewing process. After these washing processes, the malt was removed from the kettle, thus obtaining the bagasse, with a production of 100 kg of bagasse. Thirty kg of this residue was collected to obtain bagasse meal. The selected quantities were determined according to the requirements for developing and performing the physical, chemical, and structural characterization of foam trays.

### 2.2. Methodology for Obtaining Brewer’s Malt Bagasse Flour and Potato Starch

**Figure 1:**
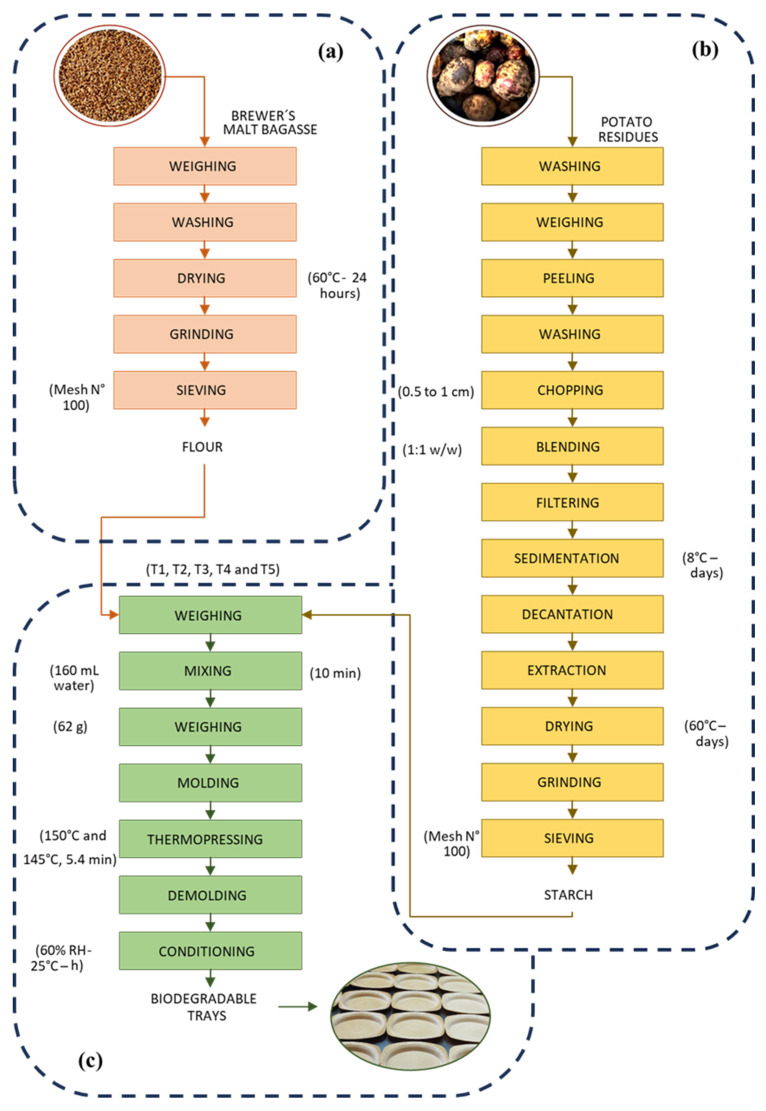
Process for obtaining (**a**) brewer’s malt bagasse flour, (**b**) potato starch, and (**c**) foam trays.

Figure 1a illustrates the methodology employed to produce brewer’s malt bagasse flour. A total of 30 kg of malt bagasse was placed on aluminum grids and subjected to a drying process in an oven (TORRH, SBT-10 × 10, Lima, Peru) at 60 °C for 24 h. After drying, the residues were ground in a hammer mill (RETSCH, ZM 200, Lima, Peru), obtaining 20 kg of material. The resulting flour was sieved through a 100-mesh sieve (CORMAC, Lima, Peru), obtaining 1.5 kg of fine flour. This flour was stored in high-density polypropylene bags until further use.

The potato residues were received directly from the fields in the town of Carabamba, and then the starch extraction process was performed by wet milling (Figure 1b) in the Microbiology and Toxicology laboratory—UNS. Thirty kg of residual potatoes were weighed using an industrial balance (PCE Instruments, model PCE-SD 150C, Meschede, Germany). The first cleaning wash was performed to eliminate impurities, such as soil and small solid particles, using approximately 40 L of potable water. Subsequently, the potatoes were manually peeled into thin strips, making sure not to lose as much pulp as possible. The peeled potatoes were then washed a second time to rinse and remove any remaining impurities. The potatoes were then cut manually into slices between 0.5 and 1 cm thick, which favored the formation of symmetrical starch granules during milling. The potato slices were placed in liquefying equipment (Oster, BLSTPEG-CPB, Hilliard, OH, USA) with a dilution of 1 L of water for each kg of potato, which allowed the release of starch through cell rupture, obtaining a milky-colored liquid suspension. The suspension was filtered through organza cloth, and approximately seven buckets of 20 L each were used to contain the resulting liquid. These buckets were stored in a refrigeration chamber at 5 °C for a period of 48 h, during which the cloudy liquid fraction, corresponding to the impurities, was separated and discarded, changing the washing water 4 to 5 times a day, until a clean starch solution free of contaminants was obtained. Once the decanting process was complete, the clean starch was removed from the buckets, spread on rectangular aluminum trays, and dried in an oven (TORRH, SBT-10x10, Peru) at 60 °C for 48 h. The yield of extracted starch was 10%. Subsequently, the dried starch was sieved through a 100-mesh sieve (CORMAC, ASTM standard, Peru). Finally, 3 kg of starch were obtained and stored in high-density polypropylene bags (Retail, MSLL 300, Lima, Peru).

### 2.3. Development of Foam Trays with Biodegradable Potential

As shown in Table 1, the foam trays were produced using different concentrations of brewer’s malt bagasse flour (0–40%) and potato starch (60–100%), resulting in five formulations. The raw materials and inputs (6% magnesium stearate, 7% glycerol, and 1% guar gum) were weighed on an analytical balance (Precisa Gravimetrics AG, LX320A, Switzerland). The magnesium stearate was used as a lubricant (release agent and anti-caking agent) helping to prevent the mixture from adhering to the mold or machinery during the thermoforming process; on the other hand, glycerol acted as a plasticizing and essential agent to give an adequate texture to the foam trays and improve their mechanical integrity. Finally, guar gum, which had binder, thickener, and film functionalities, improved the cohesion of the mixture components to help retain moisture, which is useful during thermoforming. All these reagents were obtained from Merck Laboratory (Hesse, Germany). According to the methodology indicated in Figure 1c, the formulations and the inputs were mixed in a mechanical mixer (IMACO, HM 505, Lima, Peru) for a time of 10 min. The weight of the dough for the formation of each tray was 62 g, which was placed on butter paper, to which the release agent (palm oil) was added. The thermoforming process of the foam trays was carried out in a thermopress (Reles, MS3 Digital, Lima, Peru) at a temperature of 150 °C (lower plate) and 135 °C (upper plate) for a time of 5.7 min. The foam trays were removed from the thermopress using gloves and tongs, and the final product was placed in a clean environment with a temperature of 25 °C and relative humidity of 60% for 5 h. Finally, the foam trays were stored in polypropylene bags (Retail, MSLL 300, Peru) until further analysis.

### 2.4. Chemical Characterization of Raw Materials

The BMBF and PS samples were chemically characterized. The moisture percentage was determined using the oven (POL-EKO-Aparatura, SLW 115STD, Wodzislaw Slaski, Poland), according to AOAC 931.04 [19]. Fat determination was carried out according to the AOAC method. 920.39 [20]. Ash was determined using the muffle (Thermolyne, 347034984, Waltham, MA, USA), at 600 °C for 2 h, according to AOAC 923.03 [21]. Crude fiber was determined according to the AOAC method 962.09 [22]. Protein content was determined using the AOAC method 920.87 [23]. The carbohydrate content was determined by difference: % carbohydrate = 100% − % moisture − % ash − % protein − % fat − % fiber. Amylose and amylopectin content were determined using the method applied by Ruiloba [24] and Hassan et al. [25]. A total of 0.1 g of starch was weighed, and 1 mL of 99% ethanol and 9 mL of 1 M NaOH were added. The solution was heated for 10 min until the starch gelatinized. After cooling, it was calibrated in a volumetric flask to 100 mL. Then an aliquot of 5 mL was extracted; 1 mL of 1 M acetic acid and 2 mL of iodine solution were added. It was made up to 100 mL in a volumetric flask, and the absorbance was read at 620 nm in a spectrophotometer (Jasco, V-670, Easton, MD, USA). The analysis was performed in triplicate, and the result was reported as % amylose = 3.6 × A × 20 and % amylopectin = 100% − % amylose. Where A: Absorbance at 620 nm.

### 2.5. Characterization of Foam Trays

The color attributes of the foam trays were assessed utilizing a colorimeter (Hunterlab, MiniScan XE147, Reston, VA, USA), based on the CIElab scale, which comprises L* for lightness, a* for redness/greenness, and b* for yellowness/blueness. The total color deviation (${∆E}^{*}=\sqrt{\left({∆L}^{*2}+{∆a}^{*2}+{∆b}^{*2}\right)}$) was determined taking into account the white background values (L* = 93.49, a* = 0.77, and b* = 1.40). For each analysis, the area of each sample was 4 × 3 cm, in triplicate. The thickness of the foam trays was measured with a hand-held micrometer (Mitutoyo, model 1402, Lima, Peru) capable of measuring within the range of 0 to 150 mm. The bulk density (g.cm^−3^) of the foam trays was calculated following the approach outlined by Aguirre et al. [26]. The moisture content of the foam trays was assessed using the oven-drying technique, where 5 g of crushed tray material was subjected to 105 °C heat for 3 h. The water absorption capacity was assessed following the procedure outlined in ABNT NBR NM ISO 535 (2014) [27]. Tensile and elongation tests were performed as described by Mello & Mali [28], using a texture analyzer (TA. HD Plus; Stable Micro System Ltd., Surrey, UK). Hardness and fracturability tests are conducted using the HDP/FSR holder positioned at the bottom of the texture analyzer. The penetration speed is set at 1 m/s, and a spherical probe P/0.5 S with a deformation distance of 15 mm is employed.

### 2.6. Fourier Transform Infrared Spectroscopy (FTIR)

The molecular structure and functional groups present in the foam trays were analyzed using Fourier Transform Infrared Spectroscopy (FTIR). Spectra were obtained with a Thermo Scientific™ Nicolet™ iS20 spectrometer (Waltham, MA, USA), equipped with a single-bounce attenuated total reflectance (ATR) accessory. Measurements were performed in the mid-infrared (MIR) range of 4000–500 cm^−1^, with a spectral resolution of 4 cm^−1^, and averaging 32 scans per sample to ensure signal clarity and reproducibility.

The FTIR-ATR technique allowed for rapid, non-destructive surface characterization, enabling identification of key chemical bonds and interactions between biopolymeric components.

### 2.7. Thermogravimetric Analysis (TGA)

The thermal stability and decomposition behavior of the foam trays were evaluated by thermogravimetric analysis (TGA) using a high-resolution thermal analyzer (TGA 5500, TA Instruments, New Castle, DE, USA). Approximately 10 mg of each sample was weighed and placed in a platinum crucible, then heated from 20 °C to 600 °C at a constant rate of 10 °C/min under a continuous flow of high-purity nitrogen gas (100 mL/min) to prevent oxidative degradation. The thermal events were recorded using TRIOS™ software (version 5.4, TA Instruments), which provided thermogravimetric (TG) curves and their derivatives (DTG). From these, key thermal parameters were extracted, such as the onset temperature of decomposition, maximum degradation temperature, and the residual mass at 600 °C, offering insights into the material’s thermal resistance and degradation profile. All analyses were performed at the Agroindustrial Research Laboratory of the Universidad Nacional José María Arguedas (UNAJMA), Andahuaylas, Peru.

### 2.8. Differential Scanning Calorimetry (DSC)

To determine the gelatinization temperature (Tg) and enthalpy of gelatinization (*EF*), differential scanning calorimetry was used (TA Instruments, DSC-2500, USA), previously calibrated with indium of 99.99% purity. The samples were analyzed in a hermetically sealed aluminum capsule, and the measurement was made by comparing with the heat flux of a similar, empty capsule (blank). The mass of the sample was 7.0 ± 0.1 mg, of which 75% corresponds to water and the remaining 25% corresponds to flour. After sealing the capsule, the sample was allowed to stand for 30 min to homogenize the mixture. Heating was carried out at a heating rate of 5 °C/min, from a room temperature of 20 °C to 200 °C, in a nitrogen atmosphere. For the execution of this analysis, the equipment was used with TRIOS^TM^ Software (TA Instruments, version 5.4, USA).

### 2.9. Statistical Analysis

Statistical analyses were performed using Minitab^®^ Statistical Software (version 18; Minitab LLC, State College, PA, USA). To evaluate differences between two independent groups, a Student’s *t*-test was applied. When more than two treatment means were compared, a one-way analysis of variance (ANOVA) was conducted, followed by Tukey’s Honest Significant Difference (HSD) post hoc test to identify specific differences among group means. All statistical tests were carried out at a 95% confidence level, and differences were considered statistically significant when *p* < 0.05. All chemical, molecular, and thermal characterization analyses were performed in triplicate.

## 3. Results and Discussion

### 3.1. Raw Material Characterization

The chemical composition of brewer’s malt bagasse (BMBF) of the Pale Ale variety is shown in Table 2. These results were similar to those reported by Mello & Mali [28], who determined a composition of 2.78% ash, 4.44% lipids, 5.34% moisture, 13.60% protein, and 73.84% carbohydrates (including fiber). The 30.15% crude fiber value reported in the present study is consistent with a high total dietary fiber content, previously reported at 63.84%, of which 61.83% corresponds to insoluble fiber [28]. It is important to note that Steinmacher et al. [29] confirmed that in malt bagasse, there is a significant presence of cellulose (11.35%), lignin (24.05%), and hemicelluloses 28.97%. According to Santos et al. [30], the variation in malt bagasse composition is due to the variety, harvest time, and conditions of the malted barley, as well as the quality and type of adjunct added during the fermentation process, which is unique for each brewery.

The PS had a low protein content of 1.27% and a low lipid content of 0.24%, indicating that the starch extraction process had effectively removed these compounds, resulting in a material with a high carbohydrate content of 89.61%. Kita et al. [31] reported a protein content of 1.81 g.100 g^−1^ dry weight and a fat content ranging from 0.17 to 0.63 g.100 g^−1^ dry weight for the yellow-fleshed potato. Alam et al. [32] reported the ash and fiber content of nine varieties of orange-fleshed sweet potato grown in Bangladesh, which were found to be 1.17–1.31 g.100 g^−1^ dry weight and 0.30–0.54 g.100 g^−1^ dry weight, respectively. Therefore, it is evident that the dry weight of carbohydrates found in the present investigation (89.61 g.100 g^−1^) is superior to that found in studies conducted by these authors, who quantified a lower carbohydrate content and a higher protein, fat, ash, and fiber content than PS. Vargas et al. [33] reported values of 9.43% moisture, 0.36% ash, 0.04% fat, 0.51% protein, 0.00% fiber, and 89.66% carbohydrate for starch extracted from yellow-fleshed potato. These results were similar to the present data, except for protein (1.27%) and fat (0.24%). On the other hand, Montoya et al. [34] reported values of 10% moisture, 87.79% carbohydrate, 1.18% protein, 0.15% lipid, 0.26% ash, and 24.35% amylose for potato starch, presenting a difference of 1.695% in moisture. These variations may be due to factors such as the growing zone and the methods used for starch extraction. The values obtained of 33.29% amylose and 66.70% amylopectin in the starch of Ismena yellow-fleshed potato are close to those reported by Martinez et al. [35] in varieties such as ‘Ccompis’, which present 32.69% amylose and 67.31% amylopectin. In addition, the study by Aguilar [36] indicates that potato starches generally contain between 20% and 30% amylose and 70% to 80% amylopectin; the differences between amylose and amylopectin can be attributed to factors such as the specific variety, growing conditions, and processing techniques used.

### 3.2. Characterization of Foam Trays

#### 3.2.1. Physical Properties

**Figure 2:**
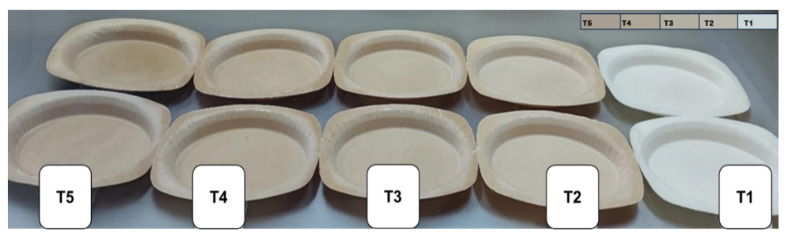
Foam trays developed from brewer’s malt bagasse flour (A) and potato starch (B) in different formulations. T1: 100%B, T2: 10%A + 90%B, T3: 20%A + 80%B, T4: 30%A + 70% B and T5: 40%A + 60%B.

The foam trays are presented in Figure 2. Regarding the color analysis presented in Table 3, a progressive darkening was observed in the samples according to the treatment applied (T1 < T2 < T3 < T4 < T5), evidenced by a decrease in the value of the L* parameter. This reduction in brightness is accompanied by an increase in the values of the chromatic parameters a* and b*, indicating an intensification in the reddish (positive a*) or greenish (negative a*), as well as yellow (positive b*) or bluish (negative b*) shades. These changes result in an increase in the total color difference (ΔE). This behavior has been reported by several authors for fiber-reinforced starch foam trays [26,28,37,38]. Several studies have shown that package color has a direct impact on product acceptance, especially in food. According to Ampuero & Vila [39], consumers associate light colors with freshness, cleanliness, and quality, while dark tones may be perceived as less attractive or less hygienic depending on the type of product. In biodegradable packaging, although consumers are willing to accept certain visual changes as indicators of sustainability, a high ∆E* may generate visual dissonance with conventional products, affecting their acceptance [40]. However, studies such as that of Mitterer-Daltoé et al. [41] indicate that, in contexts where the ecological value of packaging is adequately communicated, consumers may tolerate or even prefer natural or more rustic colors such as those generated by agro-industrial waste. This suggests that, although the increase in BMBF reduces brightness (L*) and modifies chromatic parameters (a* and b*), acceptance can be maintained if targeted to the right market and accompanied by a communication strategy that highlights the sustainability of the product.

The color alteration is attributed to the presence of lignin in the fiber; this thermal degradation of the lignin favors the darkening of the material, as has been documented in previous studies. This may be related to Maillard reactions (non-enzymatic browning) [42,43,44].

Other physical properties were presented in Table 4; for example, the moisture content of the foam trays showed three groups (T3, T4, and T5) with no significant difference (*p* < 0.05), evidencing that as the concentration of brewer’s malt bagasse flour increases, moisture values decrease. Other studies reinforce this trend. For example, Cruz [45] observed a decrease in moisture content between 9.60% and 10.96% in foam trays based on banana pseudostems. Huertas et al. [38] also reported similar decreasing values using Umari starch and corn stover.

Regarding thickness, the foam trays presented values ranging from 2.63 to 4.77 mm, showing statistically significant differences among treatments (*p* < 0.05). The increase in thickness is attributed to the incorporation of brewer’s malt bagasse meal. These values exceeded the range of 2.20 to 3.17 mm reported by Huertas et al. [38] but were lower than those found by Machado et al. [46] and Ferreira et al. [47], who recorded thicknesses of 3.30–4.60 mm and 3.50–4.21 mm, respectively. The density of the foam trays ranged from 0.15 to 0.28 g.cm^−3^, where T1 presented the highest value and T5 the lowest. These values are higher than those of polystyrene (0.041–0.06 g.cm^−3^), which highlights the structural potential of biodegradable foam trays [48]. On the other hand, the density values presented were close to those reported by Bergel et al. [49] and Machado et al. [44], who used potato starch, cassava starch, and peanut shells, respectively. The water absorption capacity ranged from 38.71 to 69.7%, reflecting a typical response of starch-based materials. Vercelheze et al. [50] and Kaisangsri et al. [51] observed that increasing fiber concentration decreased the water absorption capacity in starch-based foams, which can be explained by the chemical nature of cellulose, which is partially insoluble in water. In addition, more fiber generates more voids, increasing absorption. This feature represents a common limitation in biodegradable foam trays due to the hydrophilic nature of starch [52]. High water absorption represents a major limitation for use in food packaging applications, especially those with high moisture content. This property can compromise the structural integrity of the packaging, accelerate undesired biodegradation, and negatively affect food quality [53]. One of the most effective strategies to mitigate this problem is the application of hydrophobic coatings, such as beeswax or chitosan, among others, that act as moisture barriers [12]. In addition, it has been shown that adjusting the proportion of malt bagasse can reduce water absorption, thus improving the functionality of the material [28]. These modifications, such as hydrophobic coatings or crosslinking, are necessary to ensure the viability of the use of these biopolymers in the food sector.

It is important to note that formulations with a higher proportion of fiber (T4 and T5) show a high wettability projection due to their low density and high-water absorption, which could compromise their use in humid environments by decreasing their rigidity and losing functionality as packaging [54]. However, these characteristics could be advantageous if rapid biodegradability or short-term applications are sought, as a more porous and moist structure accelerates microbial activity [55]. Future research is recommended to evaluate this wettability property in different environmental conditions according to its applicability.

#### 3.2.2. Mechanical Properties

The mechanical properties of the foam trays are presented in Table 5. Tensile strength did not show significant differences between some formulations (*p* < 0.05), although a decreasing trend was evidenced as the brewer’s malt bagasse meal (BMBF) content increased. Formulation T2 (10% BMBF) reached the highest value (2.92 MPa), even higher than the control (T1), suggesting that small amounts of fiber can improve strength. From T3 onwards, the values decreased significantly, with T5 (40% BMBF) having the lowest strength (0.85 MPa). These results fall between those reported by Cruz-Tirado et al. [56] (1.32 MPa) and Oliveira et al. [57] (6.10–11.5 MPa), indicating that adding more than 11% fiber reduces strength, possibly due to agglomeration or breaking of starch chains during thermoforming. As for elongation, values ranged from 0.593% to 1.640%, with T1 being the most elastic. The increase in BMBF reduced elongation, especially at T5, coinciding with Cruz [45], who points out that the lower presence of amylose decreases elasticity. Nevertheless, the values obtained were higher than those reported by Cabanillas et al. [48] and Cruz-Tirado et al. [56]. Regarding stiffness, T2 presented the highest value (5.61 N), while T4 and T5 were the lowest. Although higher stiffness would be expected with more fiber, its high proportion generated more brittle structures, possibly due to lower matrix cohesion. The fracturability ranged from 2.05 mm (T1) to 3.68 mm (T2), the latter being the most ductile. These values are lower than those of polystyrene (5.54 mm), according to Cabanillas et al. [48], reflecting higher stiffness in biodegradable foam trays. According to Cruz [45] and Lawton et al. [58], high fiber concentrations reduce structural cohesion. Studies of foam trays made from banana pseudostem and cassava starch by Dilkushi et al. [59] reported tensile strength values of about 2.8 MPa and elongation at break of about 1.3%, which are comparable to our T2 formulation (2.92 MPa; 1.42%). However, the hardness values in our study (up to 5.61 N) suggest a firmer structure, potentially advantageous for applications requiring stiffness. The mechanical performance of the trays is influenced by the interaction between starch granules and the fibrous structure of brewer’s spent grain. Higher fiber content disrupts the continuity of the starch matrix, increasing porosity and reducing tensile strength, while also decreasing flexibility due to lower mobility of polymer chains. These effects are consistent with behaviors previously reported in fiber-reinforced starch-based composites [60,61].

In summary, although Formulation T2 presented higher values in tension, hardness, and fracturability, treatment T5, which contained the highest percentage of fiber (40% BMBF), showed the lowest values in most of the mechanical properties. This indicates that an excess of fiber may compromise the structural integrity of the foam trays. However, when evaluating other functional aspects, it was observed that foam trays with lower hardness, such as those of treatments T4 and T5, are more suitable for handling. In the tension and elongation parameters, all treatments showed values within acceptable ranges, comparable with previous studies, indicating the technical feasibility of these foam trays. Finally, the T2 treatment was considered the most appropriate since it meets the functional mechanical criteria and allows for the use of agro-industrial waste. This makes it a sustainable option for the production of biodegradable foam trays, contributing to the reduction of environmental impact.

However, in the T5 formulation, given their moderate mechanical properties and high-water absorption capacity (up to 69.7%), these foamed trays are more suitable for short shelf-life food packaging applications, such as dry goods, bakery, or take-out food, where exposure to moisture is limited. Regarding Formulation T2, due to their biodegradability and moderate structural strength (tensile strength up to 2.92 MPa and elongation up to 1.42%), they are also promising candidates for seedling trays or agricultural nursery containers, where a gradual degradation is beneficial after transplanting [28]. Although biodegradation rates were not directly measured in this study, the high organic content and previous evidence on starch- and lignocellulosic-based composts suggest that these materials may degrade in a few weeks to months under composting conditions [62]. Future studies are recommended to quantify biodegradation kinetics under controlled environmental conditions.

#### 3.2.3. Molecular Vibration

**Figure 3:**
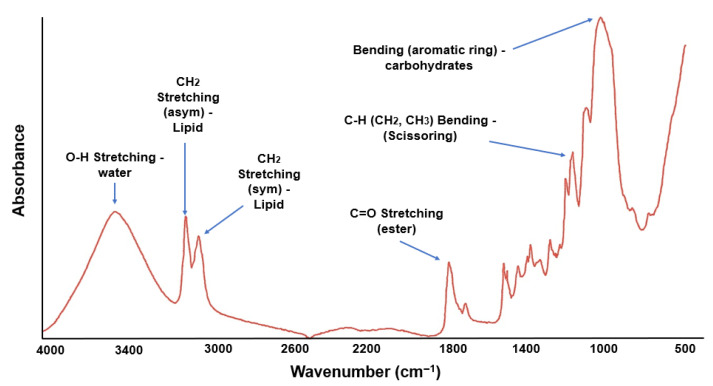
Molecular vibrational spectra measured in FTIR for foam trays of the Formulation T2 (90% potato starch and 10% brewer’s malt bagasse flour).

Figure 3 shows the FTIR spectrum of foam trays formulated with potato starch (90%) and brewer’s malt bagasse (10%). The broad band between 3200 and 3500 cm^−1^ corresponds to the O-H stretching of water, indicating the formation of hydrogen bonds with hydrophilic components such as starch and cellulose. Likewise, the band near 1645 cm^−1^, attributed to O-H stretching, reinforces this interaction, suggesting a higher affinity of the system for moisture [47,63]. In the 2800–3000 cm^−1^ region, bands corresponding to symmetric and asymmetric stretching of CH_2_ groups, associated with lipids, are observed. The low intensity of these signals is consistent with the predominantly glycidic nature of the formulation [64].

The signal near 1740 cm^−1^ is related to the stretching of the carbonyl group (C=O) of ester bonds, possibly generated by thermal reactions during the thermoforming process [47]. In the zone between 1000 and 1100 cm^−1^, the peaks at 1055 and 1027 cm^−1^ stand out, attributed to vibrations of the aromatic ring and C-O-C bonds of carbohydrates. These bands are sensitive to structural changes in starch, and their decrease at T2 with respect to the control can be explained by the lower starch concentration, adsorption by cellulose molecules, and thermally induced covalent bond breaking [63,64]. Taken together, the FTIR data suggest that the incorporation of malt bagasse modifies the molecular interactions in the polymer matrix, mainly affecting the carbohydrate functional groups and hydration, which may directly influence the physico-mechanical properties of the final material.

#### 3.2.4. Thermal Properties

**Figure 4:**
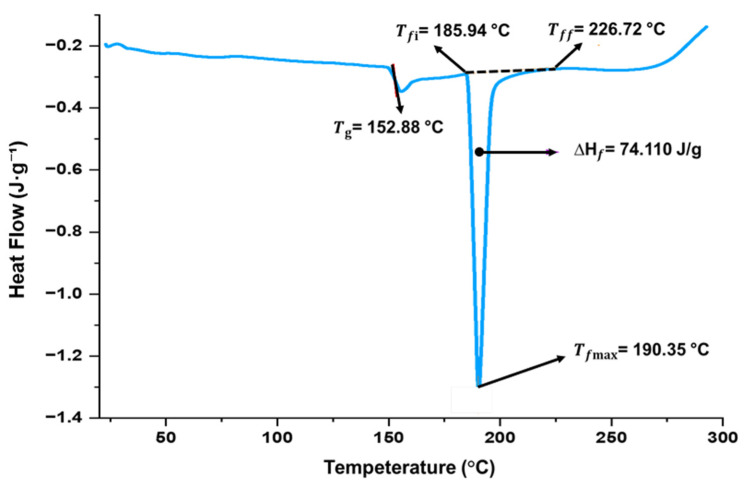
Differential scanning calorimetric analysis of foam trays of Formulation T2 (90% potato starch and 10% brewer’s malt bagasse flour).

Figure 4 shows the thermal analysis by Differential Scanning Calorimetry (DSC) for the T2 foam tray, which shows two main thermal events, evidencing endothermic peaks corresponding to the glass transition temperature (Tg) and the melting temperature. The glass transition is observed at a temperature Tg = 152.88 °C, associated with the change from the glassy (rigid) state to a more flexible state, which is attributed to an increase in the molecular mobility of the material [65]. This phenomenon directly influences the flexibility and mechanical properties of the biocomposite [66].

On the other hand, Figure 4 shows that the fusion temperature started at 185.94 °C ($T_{fi}$), reaches a peak at 190.35 °C ($T_{fmax}$), and ends at 226.72 °C ($T_{ff}$), with a fusion enthalpy (∆H_f_) of 74.110 J.g^−1^. This event represents the energy required for the material to change from a solid structure to a molten state [67]. Bustamante et al. [68] observed that hemicellulose decomposes between 180 and 220 °C, while lignin shows a more gradual degradation over a wider range of temperatures. It should be noted that, once the melting temperature is exceeded, the thermal degradation process of the material begins. These results suggest that the material possesses a high thermal stability at room temperature, as well as a high degree of thermal stability at room temperature. Regarding the measured enthalpy of fusion, it is indicative of relatively high crystallinity in the starch-based matrix. Increased crystallinity usually improves tensile strength and thermal stability but may reduce flexibility [69]. These findings are consistent with the mechanical behavior observed at T2, which shows increased tensile strength but moderate elongation, aligning with the characteristics of semi-crystalline biopolymers.

**Figure 5:**
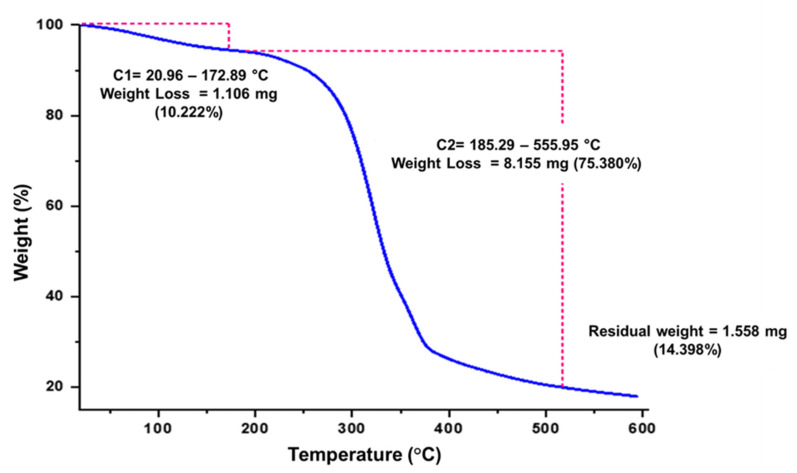
Thermogravimetric analysis in the foam tray of Formulation T2 (90% potato starch and 10% brewer’s malt bagasse flour); C1 and C2: temperature range for sample weight loss.

Thermogravimetric analysis (TGA) of the T2 tray revealed two main thermal degradation events (Figure 5). The first event, between 20.96 °C and 172.89 °C, is associated with the loss of low molecular weight volatile components, such as water, representing a mass loss of 10.22%. The second event, between 185.29 °C and 555.95 °C, represents the thermal decomposition of carbohydrates, which led to a significant loss of 75.38% of the material. The final residue (ash) reached 14.40%, indicating effective thermal resistance of the biocomposite. This behavior suggests a high thermal stability, comparable with other reinforced biodegradable materials of biodegradable origin, such as starch–fiber composites and PLA-based systems [70,71]. Comparing these results with the antecedents, it is observed that the onset temperature of the second thermal event in the T2 foam tray is lower than the maximum degradation temperature of films reinforced with Aloe vera gel and chitosan, which ranged from 293 °C to 302 °C [72]. Although T5 starts its second phase at 185.29 °C, its decomposition range extends to over 500 °C, suggesting a resilient and gradual structure in the degradation process. This contrasts with the starch–chitosan composites, in which the addition of components modified both the onset and peak of degradation. On the other hand, wheat starch films plasticized with different agents, such as fructose, glycerol, or sorbitol, showed a peak degradation temperature of up to 312.4 °C [73]. Although these values are higher in terms of peak temperature, their lower residual mass in some cases reflects more complete degradation and less final residues, which may not be desirable if higher thermal stability is sought, as occurs at T2. Studies with *Dioscorea hispida* (DH) starch reinforced with DH fiber reported peak decomposition temperatures of 329 °C, evidencing thermal improvements with the inclusion of fibers [74], similar to the effect observed in T2. The presence of fibers and lignin residues seems to be a common factor in thermal improvement.

Materials reinforced with cellulose nanofibers demonstrated stabilization of the polymer matrix by reduction in molecular mobility [75,76], a phenomenon analogous to that observed in T2. Taken together, the high residual mass and wide degradation range of the T2 tray indicate robust thermal performance, validating its potential as a biodegradable material with considerable thermal resistance. Although T2 does not yet achieve all the mechanical properties of petroleum-based plastics, its thermal characteristics and biodegradable composition position it as a promising alternative for short-term applications, especially in sustainable food packaging.

### 3.3. Perspectives of T2 Formulation in the Development of Biodegradable Trays

It is worth noting that the thermal analyses (DSC and TGA), as well as the molecular characterization, were performed exclusively on the T2 formulation, which was selected for presenting the best balance among mechanical, physical, and functional properties. This formulation was considered representative of the system due to its superior performance in terms of stiffness, tensile strength, and moisture absorption. However, it is recommended that future research expand these analyses to formulations aimed at optimizing T2. This would allow for a more accurate validation and correlation of internal structural variations with the observed functional behavior, thereby strengthening the design and optimization of biodegradable packaging based on agro-industrial waste.

Table 6 presents a comparative summary of the T2 formulation with respect to other studies. It can be seen that T2 offers a competitive combination of mechanical, thermal, and biodegradability properties, positioning itself as a balanced option compared to other starch-based formulations.

It is important to recognize the challenges of large-scale production. The use of agro-industrial wastes such as BMBF and PS significantly reduces input costs compared to expanded polystyrene, the manufacture of which relies on petroleum derivatives and is subject to market volatility [40]. However, current processes, such as thermoforming at 150 °C for almost 6 min, are still more expensive than conventional injection molding, which is faster and more efficient. Competitiveness will depend on optimizing times, automating processes, and improving thermal efficiency. With technological investment and formulation improvements—such as the use of crosslinking agents to reduce water absorption—biodegradable packaging can come closer in cost to expanded polystyrene, especially in light of increasing restrictions on single-use plastics [77].

## 4. Conclusions

This study demonstrated that the combination of 90% potato starch (PS) and 10% brewer’s malt bagasse meal (BMBF), corresponding to treatment T2, is a promising formulation for manufacturing biodegradable foam trays. This mixture adequately balanced the physical and mechanical properties, achieving a compact and stable structure. The increase in BMBF reduced moisture and density while increasing thickness and water absorption, thereby improving the cohesion of the material. In terms of mechanical properties, T2 showed effective strength and flexibility, with adequate values of hardness, fracturability, tensile strength, and elongation. FTIR, TGA, and DSC analyses confirmed interactions between the components and adequate thermal stability, evidenced by a glass transition at 152.88 °C and a high enthalpy of fusion (74,110 J.g^−1^), indicative of a partially crystalline matrix. However, practical limitations remain, such as the scalability of thermoforming for mass production and the need for uniformity in the supply of agro-industrial waste. Future work should focus on optimizing the fiber proportion within the range of 0–10% BMBF and incorporating crosslinkers to improve water resistance and mechanical durability. Overall, this formulation presents a viable and sustainable alternative to petroleum-derived plastics, supporting circular economy objectives through the valorization of agro-industrial and agricultural waste.

## Figures and Tables

**Table 1:** Formulations for the development of biodegradable foam trays from brewer’s malt bagasse flour and potato starch.

Formulation	BMBF (%)	PS (%)
T1	00.00	100.00
T2	10.00	90.00
T3	20.00	80.00
T4	30.00	70.00
T5	40.00	60.00

BMBF: brewer’s malt bagasse flour; PS: potato starch.

**Table 2:** Proximal chemical composition of brewer’s malt bagasse flour and potato starch dried (g. 100 g^−1^).

Characteristic	BMBF	PS
Moisture	4.48 ± 0.30 ^a^	8.30 ± 0.662 ^b^
Ashes	2.33 ± 0.92 ^a^	0.27 ± 0.041 ^b^
Fats	4.08 ± 0.28 ^a^	0.24 ± 0.314 ^b^
Proteins	17.99 ± 0.03 ^a^	1.27 ± 0.047 ^b^
Crude fiber	30.15 ± 0.18 ^a^	0.00 ± 0.00 ^b^
Carbohydrates	40.97 ± 0.15 ^a^	89.61 ± 0.02 ^b^
Amylose	ND	33.29 ± 0.01
Amylopectin	ND	66.70 ± 0.00

BMBF: brewer’s malt bagasse flour; PS: potato starch; ^a,b^ Different letters indicate a significant difference between the means of the samples as revealed by Tukey’s test (*p* < 0.05); ND: Not detected.

**Table 3:** Color parameters L*, a*, b*, and color difference (ΔE*) of foam trays.

Formulation	L*	a*	b*	∆E*
T1	73.07 ± 1.29 ^a^	0.03 ± 0.19 ^d^	11.49 ± 3.17 ^b^	73.68 ± 1.31 ^a^
T2	62.63 ± 1.02 ^b^	3.52 ± 0.19 ^c^	15.08 ± 0.22 ^a^	64.52 ± 0.94 ^b^
T3	56.01 ± 0.70 ^c^	4.62 ± 0.19 ^b^	15.34 ± 0.09 ^a^	58.25 ± 0.70 ^c^
T4	53.65 ± 1.18 ^c^	5.51 ± 0.14 ^a^	15.36 ± 0.15 ^a^	56.08 ± 1.20 ^c^
T5	49.11 ± 1.78 ^e^	5.80 ± 0.10 ^a^	15.52 ± 0.10 ^a^	51.54 ± 1.70 ^d^

^a–e^ Different letters indicate a significant difference between the means of the samples, as revealed by Tukey’s test (*p* < 0.05).

**Table 4:** Physical properties of foam trays made from brewer’s malt bagasse and potato starch.

Formulation	Thickness (mm)	Density (g.cm^−3^)	Moisture (%)	Water Absorption (%)
T1	2.63 ± 0.15 ^e^	0.28 ± 0.03 ^a^	4.69 ± 0.23 ^a^	38.71 ± 4.08 ^b^
T2	3.10 ± 0.10 ^d^	0.22 ± 0.00 ^b^	4.05 ± 0.13 ^b^	39.46 ± 7.45 ^b^
T3	3.60 ± 0.10 ^c^	0.23 ± 0.01 ^b^	3.51 ± 0.02 ^c^	45.39 ± 4.35 ^ab^
T4	4.00 ± 0.10 ^b^	0.19 ± 0.01 ^c^	3.51 ± 0.07 ^c^	56.70 ± 9.00 ^ab^
T5	4.77 ± 0.25 ^a^	0.15 ± 0.01 ^d^	3.42 ± 0.12 ^c^	69.70 ± 4.60 ^a^

^a–e^ Different letters indicate a significant difference between the means of the samples, as revealed by Tukey’s test (*p* < 0.05).

**Table 5:** Mechanical properties of foam trays.

Formulation	Tension (MPa)	Elongation (%)	Hardness (N)	Fracturability (mm)
T1	2.27 ± 0.56 ^b^	1.64 ± 0.26 ^a^	4.44 ± 0.32 ^ab^	2.05 ± 0.24 ^c^
T2	2.92 ± 0.23 ^a^	1.4233 ± 0.11 ^ab^	5.61 ± 0.89 ^a^	3.68 ± 0.17 ^a^
T3	2.31 ± 0.12 ^b^	1.2000 ± 0.02 ^bc^	3.72 ± 0.48 ^bc^	3.44 ± 0.34 ^ab^
T4	1.42 ± 0.15 ^bc^	1.0067 ± 0.14 ^bc^	2.99 ± 0.93 ^c^	3.08 ± 0.62 ^ab^
T5	0.85 ± 0.14 ^c^	0.5930 ± 0.276 ^c^	2.87 ± 0.49 ^c^	2.69 ± 0.73 ^bc^

^a–c^ Different letters between formulations present significant difference (*p* < 0.05).

**Table 6:** Comparison between T2 formulation and previous studies: mechanical, thermal, and vibrational properties.

Study/Material Used	Composition	Highlighted Mechanical Properties	Vibrational and Thermal Properties	Relevant Observations
Present study (T2)	90% potato starch + 10% BMBF	Tensile strength: 2.92 MPa	Elongation: 1.42%	Hardness: 5.61 N; Tg: 152.88 °C; ∆H_f_: 74.11 J.g^−1^; effective biodegradability; high thermal degradation
Cruz-Tirado et al. [52]	Yuca starch + banana pseudostem	Tensile strength: 1.32 MPa	Lower mechanical performance	Tg: ~130 °C; FTIR indicates low compatibility; lower thermal stability
Oliveira et al. [53]	Starch + natural fibers	Tensile strength: 6.10–11.5 MPa	High strength, but may imply higher production costs	TGA: thermal stability up to 200 °C; characteristic cellulose and starch vibrations present
Cabanillas et al. [44]	Starch + natural materials	Elongation: <1%	PS brittleness: 5.54 mm	T2 shows better elasticity and balanced properties
Machado et al. [40]	Yuca starch + peanut shell	Density: 0.15–0.28 g.cm^−3^	Physical properties comparable to T2	Thermal stability in the 150–180 °C range; vibrations attributed to lignin and starch
Aguilar [32]	Commercial potato starches	Amylose: 20–30%	T2 has higher amylose content (33.29%)	FTIR spectra show differences in amylose and amylopectin bands; Tg varies with composition
Bustamante et al. [64]	Biocomposites with hemicellulose	Thermal degradation: 180–220 °C	Matches the thermal degradation range observed in T2	Thermal analysis reveals stability between 180 and 220 °C; FTIR shows hemicellulose–polymer interaction

PS: potato starch; ∆H_f:_ fusion enthalpy; Tg: glass transition temperature.

## Data Availability

All the data related to this work are given here in the manuscript.

## Abbreviations

The following abbreviations are used in this manuscript:

BMBF	Brewer’s Malt Bagasse Flour
PS	Potato Starch
